# Twelve-week randomized study to compare the effect of vildagliptin vs. glibenclamide both added-on to metformin on endothelium function in patients with type 2 diabetes and hypertension

**DOI:** 10.1186/s13098-015-0062-z

**Published:** 2015-08-26

**Authors:** Luciana Neves Cosenso-Martin, Luiz Tadeu Giollo-Júnior, Débora Dada Martineli, Cláudia Bernardi Cesarino, Marcelo Arruda Nakazone, José Paulo Cipullo, José Fernando Vilela-Martin

**Affiliations:** Hospital de Base/Centro Integrado de Pesquisa da Fundação Faculdade Regional de Medicina de São José do Rio Preto (FUNFARME), São José do Rio Preto, Brazil; Internal Medicine Department and Hypertension Clinic, State Medical School in São José do Rio Preto (FAMERP), Ave Brig. Faria Lima 5416, São José do Rio Preto, SP 15090-000 Brazil

**Keywords:** Type 2 diabetes mellitus, Hypertension, Endothelial dysfunction, Arterial stiffness, DPP-4 inhibitor

## Abstract

**Background:**

Vildagliptin, a DPP-4 inhibitor widely used for the treatment of type 2 diabetes mellitus (T2DM), shows beneficial effects on endothelial function. This study aims to evaluate the effect of vildagliptin on endothelial function and arterial stiffness in patients with T2DM and hypertension.

**Methods:**

Fifty over 35-year-old patients with T2DM and hypertension, without cardiovascular disease, will be randomly allocated to two groups: group 1 will receive vildagliptin added-on to metformin and group 2, glibenclamide added-on to metformin. Biochemical tests (glycemia, glycated hemoglobin, total cholesterol, high-density lipoprotein cholesterol, triglycerides, creatinine, alanine aminotransferase, ultrasensitive C-reactive protein, and microalbuminuria), 24-h non-invasive ambulatory blood pressure monitoring, and assessment of endothelial function and arterial stiffness will be performed in both groups before and after 12 weeks of treatment. The endothelial function will be assessed by peripheral arterial tonometry, which measures the reactive hyperemia index (vasodilation), and arterial stiffness will be evaluated by applanation tonometry. All analysis will be performed using SPSS Statistical Software. For all analysis, a 2-sided *P* < 0.05 will be considered statistically significant.

**Results:**

The study started in December 2013 and patient recruitment is programed until October 2015. The expected results are that vildagliptin will improve the endothelial function in patients with T2DM and hypertension compared to glibenclamide treatment, independently of glycemic control.

**Conclusions:**

It is expected that this DPP-4 inhibitor will improve endothelial function in patients with T2 DM.

Trial registration: Clinical Trials NCT02145611, registered on 11 Jun 2013

## Background

Cardiovascular disease (CVD) is the main cause of deaths in developing and developed countries. In Brazil, CVD accounts for more than 30 % of the overall mortality rate and is responsible for 1.2 million hospitalizations/year [1]. Hypertension (HT) and type 2 diabetes mellitus (T2DM) are among the main causes of CVD. Hypertension is the most prevalent of all CVD affecting about 30–40 % of adults (over 70 million Americans and 36 million Brazilians) [2, 3]. The prevalence of T2DM ranges from 13.5 to 15 % of the population [4, 5]. As the number of elderly population is continuously growing around the world, in the next two to three decades there will be a 200 % increase in the number of individuals with ages >65 years. The prevalence of HT and T2DM is expected to increase proportionately [6].

T2DM is associated with a twofold higher risk for CVD [7]. Endothelial dysfunction is an independent predictor for future CVD in patients with T2DM [8] and is considered an early marker of vascular complications [9]. It is involved in the atherogenesis that occurs in the early stages of coronary artery disease (CAD) [10, 11]. The mechanisms of impaired endothelial function in diabetes are: reduced bioavailability of nitric oxide (NO), diminished endothelium-dependent vasodilatation, impaired barrier function, inflammatory activation, and a pro-coagulant state [12].

Today, some groups of drugs that act on the incretin system, such as glucagon-like peptide-1 (GLP-1) analogues/agonists and dipeptidyl peptidase-4 enzyme (DPP-4) inhibitors, are used to treat T2DM and may be responsible for beneficial effects on endothelial function [13].

Incretins such as GLP-1 and glucose-dependent insulinotropic polypeptide (GIP) belong a group of gastrointestinal hormones that stimulate insulin secretion in response to the ingestion of food [13]. There is evidence of physiological signaling by GLP-1 in endothelial and vascular smooth muscle cells [14–16]. GLP-1 has a vasodilator action mediated through a specific GLP-1 receptor in the vascular endothelium and may improve endothelial function in animals and humans [14, 16]. However, cardiovascular effects may be GLP-1 receptor-independent, and mediated by the metabolites of GLP-1 [17]. One study with exenatide, a GLP-1 agonist, showed a significant increase in flow-mediated vasodilatation (FMD) [18]. Moreover, liraglutide, a GLP-1 analogue, reduced plasminogen activator inhibitor 1 (PAI-1) and asymmetric dimethylarginine (ADMA) levels, thereby improving nitric oxide availability [19]. Other researchers found increases in global myocardial blood flow following GLP-1 agonist infusions in T2DM [20], and following GLP-1 infusions in healthy humans [21].

On the other hand, DPP-4 rapidly degrades incretin hormones to inactive metabolites [13] thus DPP-4 inhibitors may improve endothelial function. Studies with sitagliptin, a DPP-4 inhibitor, demonstrated an increase of endothelial progenitor cells in T2DM by inhibiting the degradation of the chemokine stromal-derived factor 1-α [22]. According to an invasive method used to measure forearm blood flow during acetylcholine infusion in T2DM patients, vildagliptin, another DPP-4 inhibitor, improved endothelium-dependent vasodilatation [23]. In contrast, other studies did not demonstrate beneficial effects in relation to the endothelium [24, 25].

Recently, three large multicenter, randomized trials testing saxagliptin [26], alogliptin [27], and sitagliptin [28] did not find any reduction in cardiovascular events, but these drugs did not increase the risk of the primary end-point either. However, the rate of hospitalization for heart failure increased in the saxagliptin arm of the SAVOR study [26]. Thus, the researchers concluded that GLP-1 inhibitors are safe for patients with CVD.

Several methods have been used to assess impaired endothelial function including plasma concentrations of markers of endothelial activity, vessel structure related to the carotid intima media thickness and arterial stiffness, flow-mediated vasodilation (FMD) and peripheral arterial tonometry (PAT).

PAT is a simple, non-invasive, and reproducible technique used to assess endothelial function [29]. Measurements of PAT in patients with CAD have been shown to strongly correlate with the parameters of endothelial dysfunction [30–33]. The Endo-PAT is an observer-independent technique that measures volume changes in the fingertip before and after blood flow occlusion and automatically calculates the reactive hyperemia index (RHI), providing an index for endothelial function.

Arterial stiffness is another parameter used to investigate endothelial dysfunction. It is recognized as a cardiovascular risk marker [34] as impaired endothelial function is one of the mechanisms involved in increased vascular stiffness [35]. Patients with both HT and T2DM exhibit increased arterial stiffness compared to those with either T2DM or HT alone [36]. Arterial stiffness parameters predict clinical outcomes (CAD, stroke, urinary albumin excretion, progression of chronic kidney disease, survival in end-stage renal disease and general cardiovascular risk) [37–42] and have a greater importance in clinical prognoses compared to other known cardiovascular risk factors such as age, gender, smoking, and dyslipidemia [43]. The non-invasive applanation tonometry technique assesses arterial stiffness by estimating arterial compliance and central blood pressure (BP) and calculates the augmentation index (AIx) [44–46]. The AIx is a marker of wave reflection derived from aortic pressure wave analysis, with increased AIx being correlated to increased stiffness and contributing to cardiovascular risk [37, 43]. Evidence shows that the central blood pressure is more relevant to cardiovascular outcomes than the BP in the brachial artery [47–49]. More recently, a study demonstrated improvement in central BP and AIx following the use of vildagliptin in a patient with T2DM and HT [50].

Thus, using the non-invasive Endo-PAT 2000 device and radial artery applanation tonometry, the purpose of this study is to evaluate the effect of vildagliptin compared to glibenclamide both added-on to metformin on endothelial function and arterial stiffness in patients with T2DM and hypertension.

## Methods

**Fig. 1:**
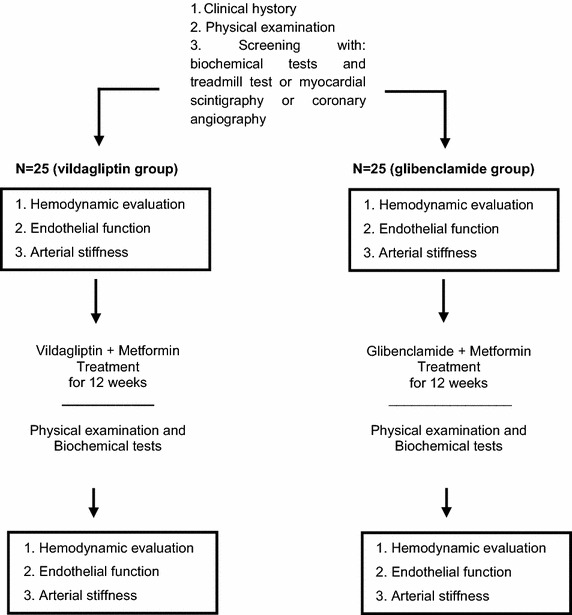
Flowchart of Study

The present trial (clinicaltrials.gov identifier: NCT02145611) will be randomized, open label, parallel assignment, controlled by drug. It was designed to assess the effect of vildagliptin (100 mg/day b.i.d.) on endothelial function in patients with T2DM and hypertension compared to glibenclamide (5–20 mg/day depending on glycemic control). The Research Ethics Committee of the institution approved the study protocol according to national and international guidelines. All patients will give their informed consent. Twenty-five individuals with T2DM and hypertension will be evaluated in the vildagliptin plus metformin group compared to 25 diabetic and hypertensive subjects in the glibenclamide plus metformin group. Figure 1 shows a flow chart of participant selection and interventions. The inclusion and exclusion criteria are presented in Table 1.

**Table 1:** Inclusion and exclusion criteria

Inclusion criteria	Exclusion criteria
Patients aged ≥35 years	Use of NPH or regular insulin, pioglitazone, GLP-1 receptor agonist, DPP-4 inhibitor or acarbose
History of DM and mild hypertension (blood pressure <160/100 mmHg) no longer than 15 years	Intolerance to metformin
Body mass index <35 kg/m^2^	Use of three or more anti-hypertensive drugs, which characterizes resistant hypertension
Glycated hemoglobin (HbA1c) between 7.0 and 10.5 %	Smoking within the previous 6 months
	Pregnancy or breastfeeding
	Creatinine clearance <45 mL/min (MDRD)
	Serum alanine aminotransferase or aspartate aminotransferase level of more than three times the upper limit of the normal range
	Subjects with ischemic heart disease, cerebrovascular disease, other atherosclerotic disease, cancer, or heart failure in functional classes II, III and IV
	Treadmill stress test with typical chest pain or with ST segment depression ≥1 mm, or with a horizontal or descending trace on the electrocardiogram for a duration of 0.08 s after the J point
	Presence of coronary heart disease diagnosed by treadmill stress test, myocardial scintigraphy or coronary angiography
	Inability to give informed consent

## Random allocation

A computer validated software (Random allocator) will be used for random allocation. The study coordinator will organize and number the envelopes which will be allocated in order of patient enrollment. The professional responsible for the Endo-PAT procedure and applanation tonometry of radial artery will be blinded.

## Randomization and follow-up

After screening for eligibility, 50 individuals will be submitted to an evaluation of endothelial function using the Endo-PAT 2000 device and measurement of the arterial stiffness by applanation tonometry of radial artery. Subsequently, they will be randomly allocated to the two arms of the study: Group 1 will receive vildagliptin (100 mg/day b.i.d.) added-on to metformin (500–2550 mg/day according to glycemic control) and Group 2 will receive glibenclamide (5–20 mg/day according to glycemic control) added-on to metformin (500–2550 mg/day according to glycemic control). Blood samples will be collected after 12-h overnight fasting at screening visit and then after 12 weeks of treatment with vildagliptin (Group 1) or glibenclamide (Group 2). Renin-angiotensin system blockers will be prescribed to all subjects at the screening visit and all other antihypertensive drugs will be maintained. Subjects will have three return consultations. The first will be to randomize patients to Group 1 or 2 and they will be evaluated after four and 12 weeks. Compliance will be monitored by pill counts at the second and last visits. The glycemic control will be evaluated by glycated hemoglobin and fasting glucose. Adverse events will be evaluated on the basis of spontaneous reports and interviews by the investigator. Considering side effects and safety, nausea, upper abdominal pain and flatulence are expected, although uncommon, while taking vildagliptin. The most frequent side effect of glibenclamide is hypoglycemic events. Changes in blood count, renal function, and liver function are not expected with either treatment.

Table 2 shows a summary of key practical aspects of the study with all follow-up visits and requested exams.

**Table 2:** Key practical aspects of the study with all the clinical visits and the requested exams

Visits	Screening	Visit 1	Visit 2	Visit 3
Weeks	−1	1	4	12
Informed consent	X			
Inclusion and exclusion criteria	X	X		
Medical history	X			
Medical evaluation/physical examination	X	X	X	X
Randomization		X		
Pregnancy test	X			
Creatinine	X			X
Fasting glucose	X		X	X
Ultrasensitive C-reactive protein	X			X
Total cholesterol	X			X
High-density lipoprotein cholesterol	X			X
Triglycerides	X			X
Glycated hemoglobin	X			X
Alanine aminotransferase	X			X
Microalbuminuria	X			X
Ambulatory blood pressure monitoring		X		X
Treadmill test	X			
Determination of the central aortic pressure and vascular stiffness markers		X		X
Endothelium function		X		X

### Anthropometric measurements

Weight and height will be measured using metric weighing scales and a measuring tape and the body mass index (BMI) will be obtained using the formula: BMI = weight (kg)/(height in meters)^2^. The waist circumference will be determined in centimeters using a measuring tape. This measurement will be carried out midway between the anterior superior iliac crest and the last rib at the end of expiration.

### Measurement of blood pressure

BP will be measured in the office using a digital sphygmomanometer according to the VI Brazilian Guidelines on Hypertension Treatment [51]. Systolic (SBP) and diastolic blood pressure (DBP) will be recorded. Hypertension will be defined as a SBP ≥ 140 mmHg and/or a DBP ≥ 90 mmHg or current use of anti-hypertensive drugs.

### Biochemical tests

Blood samples will be drawn after 12 h of fasting to measure total cholesterol (TC), high-density lipoprotein cholesterol (HDLc), triglycerides (TG), glycemia, serum creatinine, alanine aminotransferase, glycated hemoglobin and ultrasensitive C-reactive protein (CRP). Turbidimetry (BioSystems) will be used to measure the CRP and the possibility of patients having had acute infectious or inflammatory processes within recent weeks will be excluded. Microalbuminuria will also be evaluated. To evaluate microalbuminuria, the urinary albumin-to-creatinine ratio (UACR) will be obtained from urine samples collected in the morning. Urine creatinine will be calculated using a colorimetric method, and albuminuria will be determined using the nephelometric method. The glomerular filtration ratio (GFR) will be estimated using the Modification of Diet in Renal Disease (MDRD) formula: GFR_MDRD_ (mL/min/1.73 m^2^) = 186 (serum creatinine)^−1.154^ × (age)^−0.203^ × (0.742 if female) × (1.212 if black) [52].

Diabetic subjects will be identified by history of diabetes with dietetic treatment for diabetes or the use of hypoglycemic drugs. Subjects will be considered diabetics after two fasting glucose test results greater than 125 mg/dL according to the National Diabetes Data Group [53]. Serum cholesterol will be evaluated according to the Brazilian Guidelines for Dyslipidemias [54]. Low-density lipoprotein cholesterol (LDLc) will be calculated using the Friedewald formula for triglycerides levels below 400 mg/dL (LDLc = TC − HDLc − TG/5) [55].

### Evaluation of coronary artery disease

At the first (screening) visit, all selected subjects will be evaluated for CAD using the treadmill stress test. Subjects with abnormal stress test results, including typical chest pain, with ST segment depression ≥1 mm, or with a horizontal or descending trace on the electrocardiogram for a duration of 0.08 s after the J point, will be excluded. When necessary, individuals will be submitted to other tests to evaluate CAD, such as myocardial scintigraphy and coronary angiography.

### Measurement of blood pressure including 24-h ambulatory blood pressure monitoring

After randomization and again after 12 weeks of treatment with vildagliptin (Group 1) or glibenclamide (Group 2), 24-h non-invasive ambulatory blood pressure monitoring (ABPM) will be performed on a workday with a portable compact digital BP recorder (Mobil-O-Graph^®^ 24-hour PWA monitor).

Automatic BP measurements will be recorded at 20-min intervals for diurnal readings (7.00 a.m.–11.00 p.m.) and at 30-min intervals for nocturnal readings (11.00 p.m.–7.00 a.m.). Nighttime and daytime periods will be assessed based on information reported by the subjects. The sleep BP will be defined as the mean BP from the time the subjects go to bed until the time they get up. The daytime BP will be defined as the average BP during the rest of the day. The subjects will be divided into two groups according to the dip in SBP during the nighttime: participants will be considered as dippers, if the decrease in sleep SBP is ≥10 % and non-dippers if the decrease is <10 %. As part of the exam protocol, all participants will be requested to make a note of their daily activities, their meal times, times of sleeping and waking up, as well as time of taking medications and the presence of symptoms.

### Vascular tests

Vascular tests will be performed in the subjects of both groups at the first visit and after 12 weeks of treatment.

1. Determination of endothelial function

Peripheral arterial tonometry (Endo-PAT 2000; Itamar Medical, Caesarea, Israel) is a non-invasive peripheral test of endothelial function [32]. The PAT device is placed on the tip of each index finger and comprises a pneumatic plethysmograph that applies a uniform pressure to the surface of the distal finger, allowing measurement of pulse volume changes. The inflation pressure of this digital device is electronically set at 10 mmHg below DBP or 70 mmHg. The PAT signal is recorded at baseline and then after 5 min of arterial occlusion using an inflatable cuff, while the contralateral arm serves as a control. The blood pressure cuff is inflated to 60 mmHg higher than systolic pressure or at least 200 mmHg. Lack of residual pulsatility is monitored throughout the occlusion period. Post-occlusive hyperemia stimulates endothelium-dependent vasodilatation, causing an increase in digital pulse amplitude. Pulse amplitude is recorded electronically in both fingers and analyzed by an automated, computerized algorithm (Itamar Medical). The change from the baseline measurement is expressed as the RHI, which, in part, reflects vasodilator function of the digital microcirculation.

Subjects will be instructed to fast the night before testing and to refrain from ingesting alcohol or caffeine. The room temperature will be maintained between 21 and 24 °C at all times during the exam; restrictive clothing, watches, rings, or other jewelry on the hands that might interfere with blood flow will be removed. The test will be performed in the morning after the patient has been comfortably seated or has laid down in the study room for at least 15 min to reach a relaxed cardiovascular steady-state [31, 32]. The subjects of the study will be submitted to the endothelial function test at their first visit and after 12 weeks of treatment. Endothelial dysfunction will be defined as a RHI ≤ 1.68, according to a study performed in healthy asymptomatic control individuals without history of CVD and without major risk factors [56].

2. Determination of the central aortic pressure and vascular stiffness

Arterial stiffness, assessed using the non-invasive method of radial artery applanation tonometry, is predictive of vascular disease. A portion of the artery pressure wave travelling towards the extremities is reflected back by peripheral impedance points. In healthy individuals, the reflected wave returns to the aorta during diastole. However, when arteries become stiff, the transit time between the incident and reflected waves is reduced. Thus, the reflected wave returns to the aorta during systole of the same cardiac cycle thereby augmenting the central BP. This elevation of the central BP can be quantified using the AIx [57, 58]. The AIx is associated with cardiovascular risk, and predicts the presence or absence of CAD [59]. Higher values of the AIx indicate increased wave reflection from peripheral vessels or earlier return of the reflected wave as a result of increased pulse wave velocity, which is attributed to an increased arterial stiffness. In young healthy individuals, the systolic arterial pressure (aortic) is about 15–20 mmHg lower than the peripheral systolic pressure (brachial) [45, 60].

## Outcomes and outcome adjudication

1. Change in the RHI from baseline after 12 weeks of vildagliptin vs. glibenclamide treatment.
2. Change in the central blood pressure from baseline to after 12 weeks of vildagliptin vs. glibenclamide treatment.

## Statistical considerations

### Sample size and power calculations

The site http://www.lee.dante.br-pesquisa was used to estimate the sample size, considering a 30 % change in the RHI between treatment groups as clinically relevant. Assuming a standard deviation of 0.3 would require a total of 21 subjects to detect a 30 % change in the RHI with a power of 80 % at a significant level of 0.05. However, considering a potential 20 % of drop-out or lost to follow-up rate, a total of 50 patients (25 for each group) will be considered.

### Statistics

All analysis will be performed using SPSS Statistical Software (IBM SPSS Statistics for Windows, Version 21.0. Armonk, NY: IBM Corp.). Continuous variables will be presented as mean ± SD and categorical variables as frequencies. Differences between the both groups at baseline will be evaluated by unpaired *t* test or the Mann–Whitney test for comparison of continuous variables. The Chi square test or Fisher’s exact test will be employed to compare categorical variables. The change from baseline to 12-weeks follow-up in the both groups will be evaluated using the paired *t* test for continuous variables. Pearson’s correlation will be used to assess the relationship between HbA1c and RHI and AIx, after confirmation of similarity between groups in respect to demographic data (age, gender, GFR, and comorbidities: hypertension and dyslipidemia) and HbA1c targets after 12 weeks of considered treatment. Thus, the Pearson’s correlation obtained will be potentially consequent to distinct therapeutic responses. For all analysis, a 2-sided *P* < 0.05 will be considered statistically significant.

## Results/discussion

The study has started in December 2013 and patient recruitment is programed until October 2015. It is expected that vildagliptin will improve the endothelial function in patients with T2DM and hypertension more than glibenclamide treatment. Hyperglycemia causes endothelial dysfunction because it reduces the bioavailability of endothelium-derived nitric oxide (NO). Although the main action of GLP-1 is to increase glucose-stimulated insulin secretion from pancreatic beta cells, there is also evidence of physiological signaling from GLP-1 in endothelial and vascular smooth muscle cells. Thus, GLP-1 has vasodilator actions mediated by a specific GLP-1 receptor in the vascular endothelium and may improve endothelial function in animals and humans [15–17], independently of its effect on glycemic control.

Arterial stiffness is recognized as another cardiovascular risk marker [32]. Patients with both HT and DM exhibit higher arterial stiffness compared to those with either DM or HT alone [33]. Applanation tonometry estimates arterial compliance and the central blood pressure and is used to assess arterial stiffness [42, 43]. Evidence shows that the central blood pressure is more relevant to cardiovascular outcomes than the peripheral BP [44–46]. On the other hand, Endo-PAT, a non-invasive method, evaluates the vascular vasodilator propriety and, consequently, endothelial function. Both hypoglycemic drugs, vildagliptin and glibenclamide, may improve glycemic control. However, only vildagliptin and other DPP-4 inhibitors have provided beneficial effects of the endothelium [22, 23].

## Conclusions

This study will evaluate the effect of vildagliptin compared to glibenclamide, both added-on to metformin, on endothelial function and arterial stiffness in type 2 diabetic patients with hypertension. The improvement of endothelial function will demonstrate that DPP-4 inhibitors could improve cardiovascular outcome, especially in high cardiovascular risk patients.

## Abbreviations

ABPM: ambulatory blood pressure monitoring
ADMA: asymmetric dimethylarginin
AIx: *augmentation index*
BMI: body mass index
BP: blood pressure
CAD: coronary artery disease
CRP: C-reactive protein
CVD: cardiovascular disease
DBP: diastolic blood pressure
T2DM: type 2 diabetes mellitus
DPP-4: dipeptidyl peptidase-4
FMD: flow-mediated vasodilatation
GIP: glucose-dependent insulinotropic polypeptide
GFR: glomerular filtration ratio
GLP-1: glucagon-like peptide 1
HDLc: High-density lipoprotein cholesterol
HT: hypertension
LDLc: Low-density lipoprotein cholesterol
MDRD: Modification of Diet in Renal Disease
PAI-1: plasminogen activator inhibitor 1
PAT: peripheral arterial tonometry
PWV: pulse wave velocity
RHI: reactive hyperemia index
SBP: systolic blood pressure
SD: standard deviation
TC: total cholesterol
TG: triglycerides
UACR: urinary albumin-to-creatinine ratio
